# Legendre neural network-based computational study through hybrid particle swarm optimization for fractional unsteady flow of Sutterby fluid

**DOI:** 10.1038/s41598-026-45305-7

**Published:** 2026-04-01

**Authors:** Aimin Fatima, Muhammad Imran Asjad, Muhammad Naeem Aslam, Muhammad Bilal Riaz

**Affiliations:** 1 Department of Mathematics, University of Management and Technology, Lahore, 54770 Pakistan; 2 Center for Theoretical Physics, Khazar University, 41 Mehseti Str., AZ1096 Baku, Azerbaijan; 3 Department of Mathematics, Lahore Garrison University, Lahore, Pakistan; 4 IT4Innovations, VSB-Technical University of Ostrava, Ostrava, Czech Republic; 5 Applied Science Research Center, Applied Science Private University, Amman, Jordan; 6 Department of Computer Engineering, Biruni University, 34010, Istanbul, Turkey

**Keywords:** MHD flow, Sutterby fluid, Fractional order particle swarm, Legendre artificial neural network., Engineering, Mathematics and computing, Physics

## Abstract

This study investigates the two-dimensional unsteady flow of a Sutterby fluid over a stretching surface under no slip boundary condition with additional effects. The governing fractional model develop with non-local behavior of fluid properties are handled via machine learning approach based on Legendre artificial neural network (LANN). The proposed LANN computational scheme is employ, supported by optimization techniques including particle swarm optimization PSO, fractional order particle swarm optimization FPSO, and a hybrid PSO–FPSO. A novel approach is used to solve the fractional-order momentum equation of Sutterby fluid, accurately capturing memory effects and the physics of complex boundary conditions, including wall velocity, wall squeezing, and porous medium effects, which are not addressed in prior studies. An increase in the fractional-order parameter are found to accelerate the flow, reflecting enhance the memory effects. The permeability parameter produced an opposing effect, reducing velocity due to the resistance of the porous medium. Similarly, the squeezing parameter exhibited a dual role, negative squeezing enhanced velocity, while positive squeezing suppressed fluid motion. furthermore, the higher values of magnetic number is represent to velocity profile upward. Converging exhibits that standard particle swarm optimization converges less quickly, while fractional particle swarm optimization and the hybrid approach accelerate, optimized and stability achieved. The limiting behaviour of the fitness function scrutinize that PSO, FPSO, and the combinations of PSO–FPSO tend to Residual mean squared of $10^{-3}$, $10^{-5}$, and $10^{-6}$, respectively. These observance shows the deeply connection between non-integer formulating and better flow control values in determining fluid behavior. Generally, the non-integer order LANN method yields stability and rigorous solutions for intricate non-Newtonian fluid. The results is not only enriching the procedural knowledge of fractional order Sutterby model and also along with real life applications for food processing, bioengineering, polymer processing, and lubrication systems.

## Introduction

In nineteenth century, researchers investigated the non-Newtonian fluid dynamics in which they observed that the behaviour of fluid does not obey the Newton’s law of viscosity. In twentieth century, they introduced the depth understanding of the non-newtonian fluid behavior. Non-Newtonian fluids arises phenomena in natural like mudslides, avalanches and are intrinsic industrial systems as well as in polymer applications. Biofluids like blood, and food formulations such as milk, honey, yogurt, and ketchup includes complex constitutive relations. Different attributes are demonstrated like viscosity variations based on shear-thickening or shear-thinning, such as rod-climbing and die-swell effects, creep and relaxation was included in viscoelastic effects, and transient behavior studied by prior study^1^. A Sutterby fluid is a type of non-Newtonian fluid that explored the high-polymer aqueous solutions and other complex behaviour of fluids that include the shear-thinning/thickening effects. Its applied in the polymer melt manufacturing industry. Nadeem et al.^2^ analyzed the non linear, two dimensional Sutterby nanofluid through a flexible-wall channel with peristaltic flow. To find the approximate solutions applied the Homotopy Perturbation Method. This work explored on the fluid properties, bend curvature, and internal heat generation impacts in flow structure. Hayat et al.^3^ scrutinized the specific Sutterby fluid flow within a vertical channel, elaborating on the influence of a magnetic field and flexible boundaries. Shooting method was applied for establish the computational modeling by using Mathematica, that behaviour shows the effects of velocity and energy profile enhanced. Additionally, radiation heat transfer decreased the fluid’s temperature.

Ahmad et al.^4^ determined dual stratification, chemical reactions, and radiation change the heat and mass transfer within a squeezing Sutterby fluid. The Homotopy Analysis method analyzed the fluid’s behavior and the heat/mass transfer process. Results indicates the increased stratification or stronger chemical reactions decreased the heat and mass transfer rate. Brenner et al.^5^ explored that machine learning significantly enhance fluid mechanics, propelled by its expanding application and the growing availability of accessible tools. Like all computational or experimental techniques, approaches have advantages and limitations, which are acknowledged. The possibility of substantial transformation impact exists, provided that findings satisfy the established, critical standards required for fluid behavior analysis. Mir et al.^6^ applied stretching surface of a Sutterby nanofluid effects of a slanted magnetic field and temperature gradients. This model gives knowledge of the fluid behavior, thats more precise terminology in polymer reaction engineering. Nawaz et al.^7^ scrutinized a intricate modelling to analysis the thermal effectiveness of monoethylene glycol rises with $\mathrm {MOS_{2}}$ and $\mathrm {SiO_{2}}$ nanoparticles. Mixtures of these nanoparticles increasing the flow velocity near surfaces, decaying resistance to thermal transport. They rugosity this composite fluid against a traditional fluid, establish a significant enhance in thermal energy extraction. This results validation of the composite fluid’s enhanced the heat transfer rate, representing advanced results in forced/natural convection system design.

Bilal et al.^8^ established the mass and heat transfer in Sutterby fluid through a magnetic flux for the Darcy-Forchheimer coefficient. Cylindrical flow equations was represented in the intricate non-linear system. Scaling transformation was used to form a dimensionless equations. Numerical and graphical methods shows the influence of different parameters on flow attributes and boundary effects. Sabir et al.^9^ introduced the impacts of a tilted magnetic field and heat of Sutterby fluid. The magnetic field rises velocity and declines the heat transfer profile, initially enhanced the flow of the thermal state behaviour of the fluid. This research represented the understanding of these parameters in industrial and civil engineering. Imran et al.^10^ explored biological principles of the hydrodynamic forcing including the Sutterby fluid, examining mass and heat transfer through chemical processes. Perturbation method was used to solved the reduced equations, focusing on thermal and elastic structural. Higher elasticity rises flow behaviour, but reactions have an inverse impacts the concentration. Viscous dissipation increasing temperature, whereas increased effective resistance enhanced the friction forces. This research was relevant to biomedical micro-pumps, drug delivery, tissue engineering and medical devices. This paper^11^ showed the peristaltic flow of Sutterby fluid in a symmetric channel with thermal effects. The study analyzed mass and heat transfer under low Reynolds number and long wavelength assumptions. The analysis generated solutions for velocity, heat transfer rate, and concentration for minimum values of the Sutterby fluid parameter. Graphical results illustrate the impacts of distinct key variables, highlighting the effects of emerging parameters.

Mehmood et al.^12^ represented the biologically inspired computational techniques that handle the initial value problems within nonlinear RL circuits. The approach continuously combine feed-forward artificial neural network technique, sequential quadratic programming, and genetic algorithm global exploration for local refinement. The Sutterby fluid is an essential rheological model of representing both shear-thickening and shear-thinning behaviors. Boundary-layer flow over a stretched surface is of considerable industrial relevance in extrusion and sheet-forming processes. Classical integer-order formulations often fail to capture unsteady flow behavior with memory effects, motivating the use of fractional calculus in fluid-flow modeling by Sogbetun et al.^13^. Sabir et al.^14^ introduced a numerical technique to address nonlinear second-order Lane-Emden-pantograph delay differential equations. It employed artificial neural networks, training with a combined genetic algorithm and active-set method. Developers construct an objective function tailored to the Lane-Emden-pantograph equation using mean square error and ANN continuous mappings. Chou et al.^15^ introduced an enhanced fractional-order evolutionary swarm optimizer to address the limitations of conventional swarm algorithms, including inadequate accuracy in high-order problems, premature convergence, and lack of robustness. The proposed FO-PSO with non-local behavior, where position updates including both previous and current generations, showing more stability and reliability. Results represented that FO-PSO attained exceptional stability, robustness, and reliability in accuracy level.

The field of computational fluid analysis has accomplished fast improvement for the integrating massive datasets from different techniques, including experiments, simulations, and real-world experiments. Accordingly, artificial neural network are becoming standard framework in engineering and computational science, enabling valuable understanding from data capturing through accessible software. Researchers analyzed and classification of approachable algorithms, demonstrating their efficiency and precision in addressing fluid-relevant challenges. Studies of fluid datasets demonstrate that non-linear, decision tree-based algorithms consistently perform exceptionally well in replicating fluid properties by Sofos et al.^16^. Li et al.^17^ analyzed the optimized flow of Sutterby nanofluid over a stretched surface under the influence of magnetic field, radiation, and external heat sources. Thermophoresis, Brownian motion, and first-order chemical reactions was incorporated into the model. The nonlinear governing equations was solved numerically, and the effects of major flow parameters illustrated graphically. Results reveal that velocity rises with Reynolds and Deborah numbers, concentration decreases with higher Schmidt number, and entropy generation increases with Reynolds number and Brinkman parameter. Abbas et al.^18^ explored the Sutterby nanofluid over a non-uniform stretching cylinder with magnetic, thermal, and chemical effects using numerical methods. Results show that temperature decreases with higher Prandtl number, while viscous dissipation that are Eckert number enhances thermal energy. Ahmad et al.^19^ analyzed the fractional Casson fluid’s flow between parallel plates. They transformed the governing equations using integer order Fourier’s and Fick’s laws and solved. This study the parameter effects on fluid properties and also shows the effects of different trends between velocity and resistance, associate for transport of thermal energy. They gives the solutions of fractional model which increased the relative capabilities. By^20^ researchers comprehensively studied the non-integer Casson fluid’s modelling between squeezing parallel surfaces, including the impacts of porous material and magnetic fields. They transformed the constitutive equations through a similarity transformation and represents the models validity by comparing its outputs with prior results. A strengthened computational techniques resolved the intricate equations, representing unique parameter behaviors within the fractional modelling. Qayyum et al.^21^ scrutinized the unsteady non-integer Casson nanofluid flow between parallel surfaces. They considered magnetohydrodynamic and Darcian effects with both no-slip and slip conditions. They solved the fractional differential equation using a hybrid He-Laplace method, and their error validated and analysis against existent integer-order solutions represented the method’s faster convergence and accuracy. Graphic analysis emphasize distinct behaviors in slip as well as no-slip condition and highlighted the significance of fractional model for capturing memory efforts. Almeida et al.^22^ evaluated the Carreau–Casson nanofluid flow through exponentially extend curved surface, including for heat sources, chemical reactions, and activation energy parameters. The Runge–Kutta–Fehlberg method gives the numerical data to validate and train an artificial neural network (ANN), with error convergent in the range $10^{-3}$–$10^{-4}$, confirming its reliability for predicting velocity, temperature, and concentration profiles. The core of this research^23^ proposed the intricate effects of Casson fluid with squeezing forces through a porous medium, and trained to understand the fluid’s time-dependent behavior by angaging integer-order magnetohydrodynamics. Masood et al.^24^ investigated an intelligent framework for the optimal control of the Lorenz model by combining Legendre Neural Networks (LENN) with hybrid stochastic optimizers, namely the Firefly Algorithm (FA) and Archimedes Optimization Algorithm (AOA). The developed LENN-FA-AOA approach optimize network hyperparameters and tested under multiple scenarios with varying input intervals and step sizes. Numerical findings report absolute errors in the range of $10^{-5}$–$10^{-7}$, demonstrating high precision and reduced computational cost. Validation through graphical simulations, TIC and mean square error, analysis further confirms the reliability and robustness of the proposed solver. Singh et al.^25^ scrutinized hybrid deep learning framework that integrates Particle Swarm Optimization with Convolutional Neural Networks (CNNs) and Recurrent Neural Networks (RNNs) for heart disease classification. CNN architectures including $\textrm{VGG16}$, $\textrm{VGG19}$, and $\textrm{ResNet50}$ was employed, while PSO was applied for hyperparameter tuning to enhance diagnostic accuracy from CT images and patient records.

Kumar et al.^26^ established the Artificial neural networks (ANNs) effects for modeling complex nonlinear heat transfer and fluid flow problems with high accuracy and reduced computational cost. ANN methodology was applied to study mixed convective, unsteady micropolar fluid flow in a microchannel with magnetic field and activation energy using Buongiorno’s model. The ANN predictions closely agreed with numerical results, demonstrating its reliability for capturing complex transport phenomena. Kumar et al.^27^ considered the Artificial neural networks (ANNs) modeled time-dependent Casson fluid flow with non-linear radiation and magnetic effects, trained on finite difference solutions, achieving high accuracy (MSE $10^{-12}$–$10^{-8}$, absolute error $10^{-4}$–$10^{-5}$), confirming their reliability for complex flow prediction. Haider et al.^28^ introduced Non-Newtonian fluids exhibit complex rheological characteristics that cannot be described by classical Newtonian models, particularly in polymer processing and coating applications. In this work, an unsteady Sutterby fluid flow over a stretched surface was formulated by incorporating a Caputo fractional-order derivative in the momentum equation. The governing equations was reduced into a system of dimensionless ordinary differential equations using appropriate similarity transformations and solved subject to physically realistic no-slip and far-field boundary conditions, providing a generalized framework consistent with recent advances in fractional non-Newtonian fluid dynamics. Hussain et al.^29^ considered Pyramidal fins enhance heat dissipation due to their tapered design and efficient thermal performance. This study uses a physics-informed neural network (PINN) to model unsteady heat transfer in pyramidal and rectangular fins, considering convection, internal heat generation, and temperature-dependent thermal conductivity. Results show excellent agreement with finite difference solutions for the key parameters significantly affecting the heat transfer rate such as, a 17.64% increase in the convective-conductive parameter raises the heat transfer rate by 21.49%. Rawat et al.^30^ scrutinized the steady three-dimensional Newtonian and non-Newtonian ternary nanofluid flow over a bidirectionally stretching surface using Maxwell and classical fluid models. The effects of Cattaneo–Christov heat flux, magnetic field, and thermal radiation was examined, and the Nusselt number predicted using bvp4c generated data with ANN and FPSO techniques. The results show enhanced heat transfer, particularly for the non-Newtonian ternary nanofluid. Yaseen et al.^31^ established the heat transfer and flow characteristics of TiO$_2$/ethylene glycol nanofluid between horizontal coaxial tubes under the combined effects of thermal radiation and an applied magnetic field, while accounting for nanoparticle aggregation. A comparative analysis was conducted for cases with and without aggregation by examining key dimensionless parameters. Bartwal et al.^32^ considered the flow and heat transfer characteristics of a ternary hybrid nanofluid (Al$_2$O$_3$–graphene–CNT/water) over a rotating disk using a mathematical formulation combined with soft computing techniques. The effects of buoyancy force, thermal radiation, and Hall current were examined, while numerical solutions obtained through the bvp4c method were utilized to train ANN and FPSO models for accurate prediction of the Nusselt number. The results demonstrated enhanced thermal performance and high prediction accuracy. Karthik et al.^33^ investigated the Soret and Dufour effects on Casson ternary hybrid nanofluid flow past a revolving sphere in a porous medium under a magnetic field. Heat source/sink, thermal radiation, and chemical reactions influence mass and heat transfer, modeled via similarity transformations and solved using RKF-45. ANN predictions show that porous and magnetic effects reduce velocity, heat source/radiation and Biot/Dufour numbers enhance temperature, and chemical reactions lower concentration

Recent research on Sutterby and other non-Newtonian fluids has mostly relied on classical numerical schemes and integer-order formulations to study flow, heat, and mass transfer characteristics. However, such models fail to capture the inherent memory and hereditary features of realistic non-Newtonian systems. To address this limitation, the present study develops a fractional-order Sutterby fluid model using the Caputo fractional derivative to represent memory effects accurately. The problem is solved through an Artificial Neural Network (ANN) optimized via a hybrid Particle Swarm Optimization–Fractional Particle Swarm Optimization (PSO–FPSO) algorithm. The combination of fractional-order modeling with Legendre-based neural networks allows for accurate learning of complex flow patterns while preserving memory effects. This combined method improves prediction accuracy over conventional neural networks or fractional models, particularly in flows shows non-integer or memory-dependent behavior. This hybrid method accelerate learning accuracy, convergence and capability by compared with prior study. The novelty of this work lies in hybrid fractional-order modeling with an optimized ANN method, emerging in improved stability, precision, and physically reliable results.

**Fig. 1:**
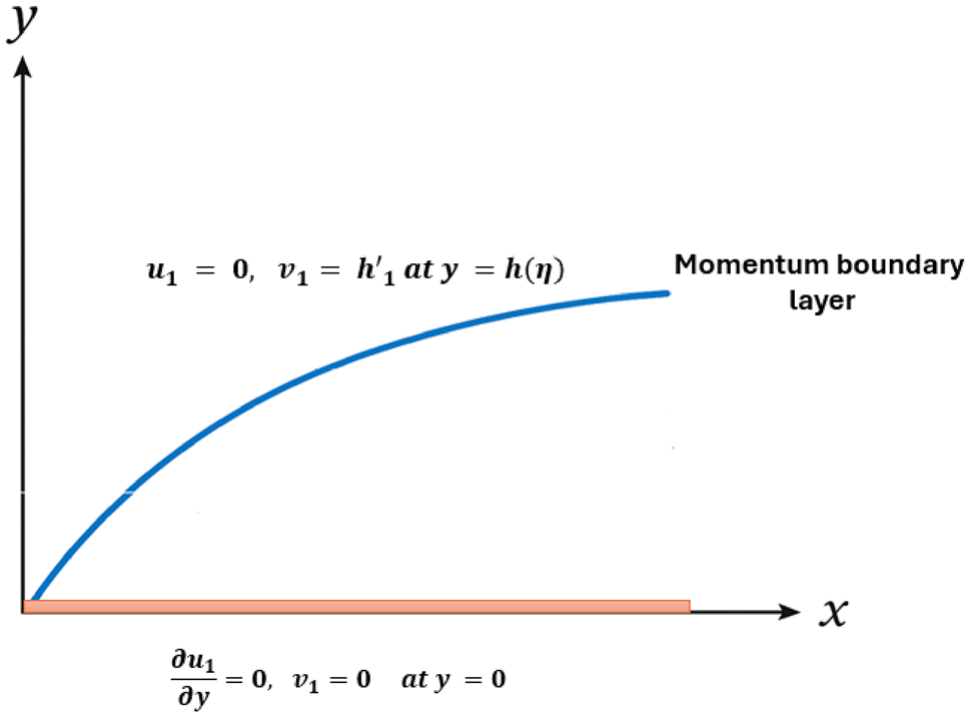
Geometry of the problem.

## Mathematical preliminaries

### Definition

The fractional derivative (FD) of real order $\alpha > 0$ for a function $\Theta (h)$, in the classical Caputo sense, is defined as^13^

1 $$\begin{aligned} D_{h}^{\alpha }\left[ \Theta (h) \right] ^{c}_{0} = \frac{1}{\Gamma (n-\alpha )} \int _{0}^{h} \frac{\Theta ^{(n)}(\xi )}{(h-\xi )^{\alpha -n+1}} \, d\xi , \quad n-1 < \alpha \le n,\; n \in \mathbb {N},\; \Theta \in C^{n}[0,1], \end{aligned}$$

where, $\dfrac{1}{(h-\xi )^{\alpha -n+1}}$ represents as the singular kernel.

### Mathematical approach for problem formulation

The two-dimensional, time dependent flow of Sutterby fluid over a stretched porous surface under the effects of a magnetic field. The flow occurred between two originally separated sheets of distance $\delta$, where the gap at any instant $\eta$ is defined as $h_{1}(\eta )=\delta (1-\gamma \eta )^{\tfrac{1}{2}}$. Here, $\gamma$ shows the retreating or compressing strength of the plates, when $\gamma <0$ are retracting, while for $\gamma >0$ the sheets are compressing. The nonlinear fluid flow behaviour captures its shear-dependent, thickening and thinning effects, while the magnetic force shows to resist objects to the motion. Additionally, the drag effects of the porous medium are incorporated to reflect realistic physical conditions. At the surface, the fluid satisfies the no-slip condition, while the vertical velocity accounts for wall squeezing to represent the motion of the boundary. Away from the surface, the flow approaches the free-stream, where the velocity gradient becomes zero, indicating that the fluid remains undisturbed. These combined influences highlight the interaction of stretching flow, magnetic field, and porous resistance in controlling Sutterby fluid behavior. Eq.(2) defining the Cauchy stress tensor ${\tau }$.

2 $$\begin{aligned} {\tau } =-p{{{\bf I}}}+{{{\bf S}}}, \end{aligned}$$

For Sutterby fluid model, defining extra stress tensor **S** is defined in Eq. (3) as follows^21^.

3 $$\begin{aligned} |{\gamma }| = \sqrt{\frac{1}{2}trace ({{{\bf A}}_{1}})^2},\ \ \ {{{\bf A}}_{1}} = {{{\bf L}}}+{{{\bf L}}^T}, \ \ \ {{{\bf L}}}= {\nabla {{\bf V}}}, \ \ \ \textrm{S} = {\mu _{0}}\left( \frac{\sinh ^{-1} {{\bf B}}\sqrt{|{\gamma }|^2}}{{{\bf B}}\sqrt{|{\gamma }|^2}} \right) ^m |{\gamma }|, \end{aligned}$$

where, $\mu _{0}$ represents the dynamic viscosity of the fluid, whereas *B* denotes the characteristic time or fluid parameter. The symbol *m* stands for the power-law index, and $\dot{\gamma }$ indicates the second invariant of the strain-rate tensor. Here, ${\bf A}_{1}$ refers to the first Rivlin–Ericksen tensor, and ${\bf V}$ is the velocity vector.

The momentum equations are given below from^21^ as shown in figure 1.

4 $$\begin{aligned} \frac{\partial u_{1}}{\partial \eta }+v_{1}\frac{\partial u_{1}}{\partial y}+u_{1} \frac{\partial u_{1}}{\partial x}= & \frac{\mu }{\rho }\frac{\partial }{\partial y } \left[ \frac{\partial u_{1}}{\partial y}+\frac{mB^2}{2}(\frac{\partial u_{1}}{\partial y})^3\right] -\frac{\sigma B_{0}^2}{\rho }u_{1}-\frac{\mu }{\rho K^*}u_{1}, \end{aligned}$$

5 $$\begin{aligned} \frac{\partial v_{1}}{\partial \eta }+u_{1}\frac{\partial v_{1}}{\partial x}+v_{1}\frac{\partial v_{1}}{\partial y}= & \frac{\mu }{\rho }\frac{\partial }{\partial y}\left[ \frac{\partial v_{1}}{\partial y}+\frac{mB^2}{2}(\frac{\partial v_{1}}{\partial y})^3\right] -\frac{\mu }{\rho K^*}v_{1}, \end{aligned}$$

The boundary conditions are given below^13^,

6 $$\begin{aligned} u_{1}= & 0,\ \ v_{1}=h_{1}', \ \ at\ y=h(\eta ),\end{aligned}$$

7 $$\begin{aligned} \frac{\partial u_{1}}{\partial y}= & 0,\ \ v_{1}=0, \ \ at\ y=0. \end{aligned}$$

The following non-dimensional coordinates are introduced^13^, into Eqs. (4)–(7) and after simplification and by eliminating pressure terms, we have

8 $$\begin{aligned} \frac{\partial \Omega }{\partial \eta } +u \frac{\partial \Omega }{\partial x}+ v \frac{\partial \Omega }{\partial y}= & \frac{\mu }{\rho }\frac{\partial }{\partial y}\left[ \frac{\partial \Omega }{\partial y}+\frac{mB^2 }{2}\left( \frac{\partial w}{\partial y}\right) ^3\right] -\frac{\mu \Omega }{\rho k^*}+ \frac{\sigma B_{0}}{\rho } \frac{\partial u}{\partial y}, \end{aligned}$$

where,

9 $$\begin{aligned} \Omega= & \left( \frac{\partial v}{\partial x}-\frac{\partial u}{\partial y}\right) , \end{aligned}$$

The similarity transform are given below,

10 $$\begin{aligned} u_{1}=\frac{\gamma x}{2(1-\gamma \eta )}V'(\xi ),\ \ v_{1}=\frac{-\gamma \delta }{2(1-\gamma \eta )^{\frac{1}{2}}}V(\xi ),\ \ \xi ={\frac{y}{\delta (1-\gamma \eta )^{\frac{1}{2}}}}, \end{aligned}$$

which converts the governing equation into the following form.

11 $$\begin{aligned} \left( 1+\frac{m B^2}{2}\right) \frac{d^4 V}{d \xi ^4}- M_{P}\frac{d^2 V}{d \xi ^2}-M_{g}\frac{d^2 V}{d\xi ^2}- S_{q}\left( \xi V (\xi )+ 3 \frac{d^2 V}{d \xi ^2}+ \frac{d V}{d \xi } \frac{d^2 V}{d \xi ^2}-V \frac{d^3 V}{d \xi ^3}\right) =0, \end{aligned}$$

where, $M_{p}= \frac{\mu \delta ^2 (1-\gamma \eta )}{\nu k \rho },$ and $M_{g}=\frac{\sigma B^2 \delta ^2 (1-\gamma \eta )}{\nu \rho },$ are the permeable factor and magnetically charged, respectively. $S_{q}=\frac{\gamma \delta ^2 }{2 \nu }$ is the non-dimensional Squeeze number, this number $S_{q}$ describes the movement of the plates $S_{q}>0$ corresponds to the plates moving apart, while $S_{q}<0$ corresponds to the plates coming together.

The time fractional model of Eq. (11) is as follows, where $D_{\eta }^\alpha$ represents Caputo fractional operator and defined in Eq. (1)

12 $$\begin{aligned} z \left( 1+\frac{m B^2}{2}\right) D_{\eta }^\alpha V- M_{p}\frac{d^2 V}{d \xi ^2}-M_{g}\frac{d^2 V}{d\xi ^2}- S_{q}\left( \xi V(\xi )+ 3 \frac{d^2 V}{d \xi ^2}+ \frac{d V}{d \xi } \frac{d^2 V}{d \xi ^2}-V \frac{d^3 V}{d \xi ^3}\right)= & 0, \end{aligned}$$

where, the possible values of the fractional parameter is $\alpha \in (3,4]$.

The boundary constraints now take the form,

13 $$\begin{aligned} V(0)=0,\ \ V''(0)=0, \ \ \ V'(1)=0,\ \ V(1)=1.\ \ \ \end{aligned}$$

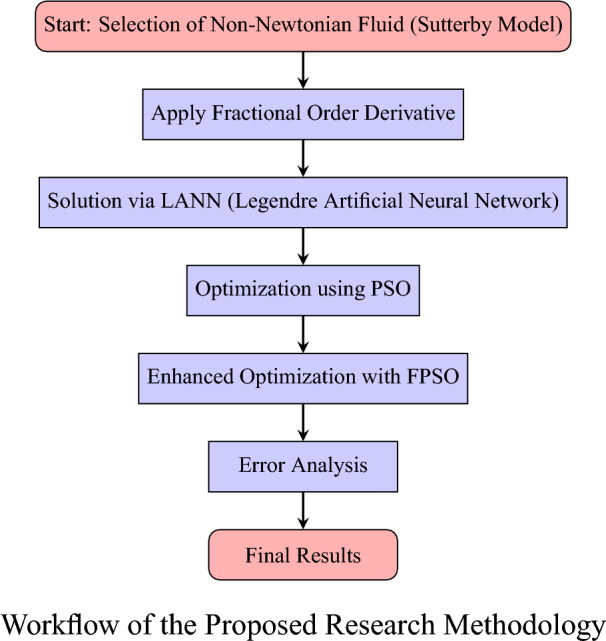


## Solution methodology

In machine learning principles are integrated by distinguishing between supervised and unsupervised learning strategies. Supervised learning employs labeled datasets with known input–output pairs, allowing the model to learn explicit mappings and make reliable predictions. Additionally, unsupervised learning without focusing of disclosure hidden structures, labeled outputs, relationships or clusters within the data. Artificial Neural Networks (ANNs) represents different techniques like as radial basis networks, feed-forward method, recurrent structures, and convolutional designs, each deeply used for tackled intricate and nonlinear problems. However, these conventional networks often includes limitation such as risk of trapping in local minima, slow training speed and inefficiency for higher order non-linear systems.

The neural network approach is increased through activation functions, which introduce non-linearity and allows the network to handle the intricate systems. Common activation functions like ReLU (Rectified Linear Unit), tanh, sigmoid, and leaky ReLU are frequently used in different problem depending on the structure requirements. For this study, a Legendre Artificial Neural Network (LANN) is employed to construct the orthogonal Legendre polynomials. The LANN improved numerical stability, yields higher convergence, and faster approximation accuracy, make it effective for nonlinear models and non-integer order models in fluid dynamics. The use of LANN rises the efficiency of problem by ensuring greater numerical stability, faster convergence, and more accurate function approximation. Its orthogonal method decline the computational effort while Preserving the ability for complicated structure, making it particularly efficient for non-Newtonian and fractional-order fluid models. The training process is iterative method in which the weights and biased are change systematically until satisfactory accuracy is obtained.

### Design of the LANN model

The proposed work for single-layer Legendre Artificial Neural Network (LANN) includes with an input layer, a functional Legendre polynomials, and an output layer. Shallow are used for hidden layer, the input patterns are used to higher-dimensional space through Legendre polynomials, which are orthogonal of the interval $[-1,1]$. Table 1 lists the first ten Legendre polynomials. Higher-order Legendre polynomials are constructed recursively as follows:

$$\begin{aligned} L_{n+1}(\xi )= & \frac{1}{n+1} \left[ (2n+1)\,\xi \,L_n(\xi ) - n\,L_{n-1}(\xi ) \right] , \end{aligned}$$

where, $L_n(\xi )$ represents the *n*-th order polynomial. The mathematical model of the problem is expressed as a series solution encompassing the input, functional expansion, and output layers. The solution and its higher-order derivatives are formulated in Eqs. (14)–(18). The function $f(t)$ and its derivatives can be expressed using Legendre polynomials as:

14 $$\begin{aligned} f(\xi )= & \sum _{i=1}^{N} \gamma _i L(\alpha _i \xi + \beta _i), \end{aligned}$$

15 $$\begin{aligned} f^1(\xi )= & \sum _{i=1}^{N} \gamma _i L^1(\alpha _i \xi + \beta _i), \end{aligned}$$

16 $$\begin{aligned} f^2(\xi )= & \sum _{i=1}^{N} \gamma _iL^2(\alpha _i \xi + \beta _i), \end{aligned}$$

17 $$\begin{aligned} f^3(\xi )= & \sum _{i=1}^{N} \gamma _iL^3(\alpha _i \xi + \beta _i),\end{aligned}$$

18 $$\begin{aligned} D_{\eta }^\alpha f(\xi )= & \sum _{i=1}^{N} \gamma _iL^4(\alpha _i \xi + \beta _i), \end{aligned}$$

where, ${\gamma } = [{\gamma _{1}}, {\gamma _{2}}, {\gamma _{3}}, \ldots , {\gamma _{n}}], \; {\alpha } = [{\alpha _{1}}, {\alpha _{2}}, {\alpha _{3}}, \ldots , {\alpha _{n}}], \; {\beta } = [{\beta _{1}}, {\beta _{2}}, {\beta _{3}}, \ldots , {\beta _{n}}]$ be real-valued and bounded vectors. The function can be expressed as, where *L* denotes the Legendre polynomial, *n* represents the order of the polynomial, and *i* indicates the neuron index in the Legendre Artificial Neural Network (LANN). We choose $n=6$ that is $\tfrac{1}{16}\,(231\xi ^{6}-315\xi ^{4}+105\xi ^{2}-5)$ .

**Table 1:** First ten Legendre polynomials $L_n(\xi )$.

*n*	$L_n(\xi )$
0	$1$
1	$\xi$
2	$\tfrac{1}{2}(3\xi ^{2} - 1)$
3	$\tfrac{1}{2}(5\xi ^{3} - 3\xi )$
4	$\tfrac{1}{8}(35\xi ^{4} - 30\xi ^{2} + 3)$
5	$\tfrac{1}{8}(63\xi ^{5} - 70\xi ^{3} + 15\xi )$
6	$\tfrac{1}{16}(231\xi ^{6} - 315\xi ^{4} + 105\xi ^{2} - 5)$
7	$\tfrac{1}{16}(429\xi ^{7} - 693\xi ^{5} + 315\xi ^{3} - 35\xi )$
8	$\tfrac{1}{128}(6435\xi ^{8} - 12012\xi ^{6} + 6930\xi ^{4} - 1260\xi ^{2} + 35)$
9	$\tfrac{1}{128}(12155\xi ^{9} - 25740\xi ^{7} + 18018\xi ^{5} - 4620\xi ^{3} + 315\xi )$
10	$\tfrac{1}{256}(46189\xi ^{10} - 109395\xi ^{8} + 90090\xi ^{6} - 30030\xi ^{4} + 3465\xi ^{2})$

19 $$\begin{aligned} \hat{V}(\xi )= & \sum _{i=1}^{N}\frac{1}{a_{1}} \gamma _i \left[ a_{2}(\alpha _i \xi + \beta _i)^6-a_{3}(\alpha _i \xi + \beta _i)^4+a_{4}(\alpha _i \xi + \beta _i)^2-a_{5}\right] , \end{aligned}$$

20 $$\begin{aligned} \frac{d\hat{V}}{d \xi }= & \sum _{i=1}^{N}\frac{1}{a_{1}} \gamma _i \left[ 6a_{2}\alpha _i(\alpha _i \xi + \beta _i)^5-4a_{3}\alpha _i(\alpha _i \xi + \beta _i)^3+2\alpha _i a_{4}(\alpha _i \xi + \beta _i)\right] , \end{aligned}$$

21 $$\begin{aligned} \frac{d^2 \hat{V}}{d \xi ^2}= & \sum _{i=1}^{N}\frac{1}{a_{1}} \gamma _i \left[ 30a_{2}(\alpha _i)^2(\alpha _i \xi + \beta _i)^4-12a_{3}(\alpha _i)^2(\alpha _i \xi + \beta _i)^2+2(\alpha _i)^2 a_{4}\right] , \end{aligned}$$

22 $$\begin{aligned} \frac{d^3 \hat{V}}{d \xi ^3}= & \sum _{i=1}^{N}\frac{1}{a_{1}} \gamma _i \left[ 120a_{2}(\alpha _i)^3(\alpha _i \xi + \beta _i)^3-24a_{3}(\alpha _i)^3(\alpha _i \xi + \beta _i)\right] , \end{aligned}$$

23 $$\begin{aligned} \frac{d^4 \hat{V}}{d \xi ^4}= & \sum _{i=1}^{N}\frac{1}{a_{1}} \gamma _i \left[ 360a_{2}(\alpha _i)^4(\alpha _i \xi + \beta _i)^2-24a_{3}(\alpha _i)^4\right] ,\end{aligned}$$

24 $$\begin{aligned} D_{\eta }^\alpha \hat{V}(\xi )= & \sum _{i=1}^{N}\frac{1}{a_{1}} \gamma _i \left[ 720a_{2}(\alpha _i)^5(\alpha _i \xi + \beta _i)\right] . \end{aligned}$$

### LANN-based Fitness Function

The fitness function for the proposed scheme is formulated in terms of the mean square error (MSE) with given initial conditions, as expressed in below^23^

$$\begin{aligned} E= & e_{1} + e_{2}. \end{aligned}$$

For the nonlinear Lorenz system, the error components are defined as follows,

$$\begin{aligned} e_{1}= & \sum _{j=1}^{M} \left( \left( 1+\frac{m B^2}{2}\right) D_{\eta }^\alpha \hat{V}(\xi )- M_{p}\frac{d^2 \hat{V}}{d \xi ^2}-M_{g}\frac{d^2 \hat{V}}{d\xi ^2} - S_{q}\left( \xi \hat{V}(\xi )+ 3 \frac{d^2 \hat{V}}{d \xi ^2}+ \frac{d\hat{V}}{d \xi } \frac{d^2 \hat{V}}{d \xi ^2}-\hat{V} \frac{d^3 \hat{V}}{d \xi ^3}\right) \right) ^{2}, \\ e_{2}= & \frac{1}{4} \sum _{j=1}^{M} \left( (\hat{V}(0)-0)^2+(\hat{V}'(1)-0)^2+( \hat{V}''(0)-0)^2+(\hat{V}(1)-1)^2\right) . \end{aligned}$$

The objective of constructing these fitness functions for the velocity profiles of Sutterby fluid to determine the optimal weights and biases in the LANN model to achieve reduced error. When the error value *E* converges towards zero, the proposed approach provides a precise approximation to the exact solution of the system.

### Particle swarm optimization

The concept of particle swarm optimization (PSO) was first established by Eberhart and Kennedy^23^, was inspired by the cooperative foraging patterns of bird flocks. As a swarm intelligence-based optimization method, it models a group of particles (analogous to birds) exploring a search space that contains multiple potential solutions, represented as food sources of varying quality. The ideal solution associates with to the position of the maximum food source, represented by $P_{g}$. Each particle starts with the arbitrary position, managed by its individual skill to encountered the best solution, showed as $P_{li}$. When a particle indicates the larger solution is known by the swarm. Thus, the curve orbit path of each particle is resolved by three components its personal best solution, its previous velocity, and the global best solution attained by the swarm. In particle swarm optimization, each bird in the natural parallel is presented as a particle through the solution set. The particle position indicates the possible solution of the refined problem, and each value is associated through the fitness value. Every particle has a rate of change $V_i$ that finds its movements. This rate of change is modernize by considering three parameters, the best solution discovered by the particle itself, the current velocity and the better solution gained by the entire swarm (*gbest*). After upgrades the direction and velocity, the position of particle’s is modified accordingly. This new value is determined by objective function to scale its achievement. The new solution is change by the previous one if it is best, otherwise, the original best remains unchanged. This iteration procedure continuous enabling the swarm to stability toward the optimal solution.

### Particle Swarm Algorithm Formulation

Each particle’s position and velocity are adaptively changed using the following relations^24^,

25 $$\begin{aligned} V_i(k+1)= & V_i(k)- c_{1}r_{1}\big (p_i(k)-P_{li}(k)\big ) - c_{2}r_{2}\big (p_i(k)-P_{g}(k)\big ),\end{aligned}$$

26 $$\begin{aligned} p_i(k+1)= & V_i(k+1)+p_i(k), \end{aligned}$$

where $i = 1,2,\ldots ,m$ shows the index of particle *m* is the swarm size, and *k* represents the iteration value. Here, $V_i(k)$ indicates to the velocity vector included with the *i*-th particle, while $p_i(k)$ represents its position in the solution particle space. The term $P_{li}(k)$denotes to the best position discovered so far by the *i*-th particle (personal best), and $P_{g}(k)$ refers the best position identified by the entire swarm (global best). The parameters $c_1$ and $c_2$ are the social learning and cognitive coefficients, respectively, while $r_1$ and $r_2$ are random numbers uniformly values in the interval [0, 1]. The learning parameters $c_1$ and $c_2$ represents as increased factors that control the balance between collective cooperation and individual exploration. Values are very small, particles shows to explore the vicinity of the region more thoroughly, increasing the parameter shows the optimal solution but the expense of additional computational effort. In contrast, increasing values allows the particles to converge more increasing to the global or personal best solution, so if optimized the time but the higher risk of untimely convergence. Special cases enhanced when either $c_1$ or $c_2$ is approach to zero, producing virous swarm behaviors.

In Eq (19), the first component represents to the inertia values, indicated the tendency of a particle effect to preserve its previous velocity. The second component shows the cognitive term, which introduced the self-exploration based on the particle’s. The third component refers the social term, which models the sharing of information among particles to enhanced the cooperative search. A linear combination of these three factors governs the update of the particle’s velocity and position, and new particle positions are evaluated using the fitness function, which guides the optimization procedure.

### Fractional-order particle swarm algorithm

Fractional order extends the ideas of traditional calculus. The Grünwald–Letnikov (GL) definition of a non-integer derivative $\alpha$ is represents as below^24^,

$$\begin{aligned} D^{\alpha }[x(t)]= \lim _{h \rightarrow 0} \frac{1}{h^{\alpha }}\sum _{k=0}^{\infty } (-1)^k \frac{\Gamma (\alpha +1)}{\Gamma (\alpha -k+1)\Gamma (k+1)} \, x(t-kh), \end{aligned}$$

where, $D^{\alpha }$ shows the non-integer order operator, $\Gamma (\cdot )$ indicated the Euler gamma function, and $\alpha$ represents the order, When the step size *h* refers a discrete parameter, the fractional order derivative can be written as,

$$\begin{aligned} D^{\alpha }[x(t)]=\frac{1}{T^{\alpha }}\sum _{k=0}^{r} (-1)^k \frac{\Gamma (\alpha +1)}{\Gamma (\alpha -k+1)\Gamma (k+1)} \, x(t-kT), \end{aligned}$$

where, *r* shows the truncation order and *T* represents the sampling period. Unlike the integer-order derivative, which showed the finite series, the fractional-order models includes the infinite series expansion. This characteristic enables fractional calculus to capture more comprehensive information compared to its integer-order counterpart. Moreover, incorporating fractional order change into the particle swarm algorithm shows a memory effect, where the velocity vector is combined behaviour of both effects such as the current and previous positions. As a result, the strategy based on fractional order model particle swarm includes more conservative, producing solutions that are comparatively more stable and consistent through iterations. For instance, when $r=4$, the update velocity and position equations can be written as,

$$\begin{aligned} V_i(k+1)= & V_i(k) + c_{1}r_{1}\big [P_{li}(k)- \frac{\alpha }{6}(1-\alpha )(2-\alpha )p_i(k-2) - \frac{\alpha }{2}(1-\alpha )p_i(k-1) \\ & -\alpha p_i(k)- \frac{1}{24}p_i(k-3)(1-\alpha )(2-\alpha )(3-\alpha )\big ]- c_{2}r_{2}\big (p_i(k)-P_g(k)\big ),\\ p_i(k+1)= & V_i(k+1)+p_i(k), \end{aligned}$$

where, the additional values appear from the truncated fractional order derivative for $r=4$. When the inertia weight is maximin, the updated velocity represents more optimal, smooth direction and enhanced global explored ability more than prior studies. However, if the inertia weight becomes very large, it may cause overcorrection and decrease the efficiency of the study. When the particle speed is extremely large, the motion of path may overshoot and leading to unstable the behaviors. On the other hand, when the inertia weight is too small, the velocity changes by mainly governed by the local best and global best solutions. This setting improves local search ability but limits global exploration, increasing the chance of being trapped in a local optimum. As iterations progress, the inertia weight gradually decreases, allowing the swarm to transition from global exploration to local exploitation in the vicinity of the best solutions found so far. This adjustment mechanism is described by the following equation. The transformation of the constant inertia weight into a time-varying linear inertia weight $\omega (k)$ is given below.

$$\begin{aligned} \omega (k) = \omega _{\min } + \frac{(\text {iter}_{\max } - k)(\omega _{\max } - \omega _{\min })}{\text {iter}_{\max }}, \end{aligned}$$

where $\omega _{\max }$ denotes the maximum inertia weight, *k* is the iteration counter, $\omega _{\min }$ denotes the minimum inertia weight, and $\text {iter}_{\max }$ specifies the total number of iterations. To avoid excessive velocity exceeding the search space during the particle update, Eberhart and Shi introduced the maximum velocity method to limit particle velocity. The value of $V_{\max }$ must be chosen carefully, if it is too large, particles may fly out of the search domain, if it is too small, particles will move too slowly and fail to explore the global search space. The velocity update using this method is defined as,

$$\begin{aligned} V_i(k+1) = {\left\{ \begin{array}{ll} V_{\max }, & V_i(k+1) > V_{\max }, \\ -V_{\max }, & V_i(k+1) < -V_{\max }, \\ \end{array}\right. } \end{aligned}$$

$V_{\max }$ is set to be 0.2 times the maximum search range.

$$V_{\max } = 0.2 \, X_{\max }.$$

**Fig. 2:**
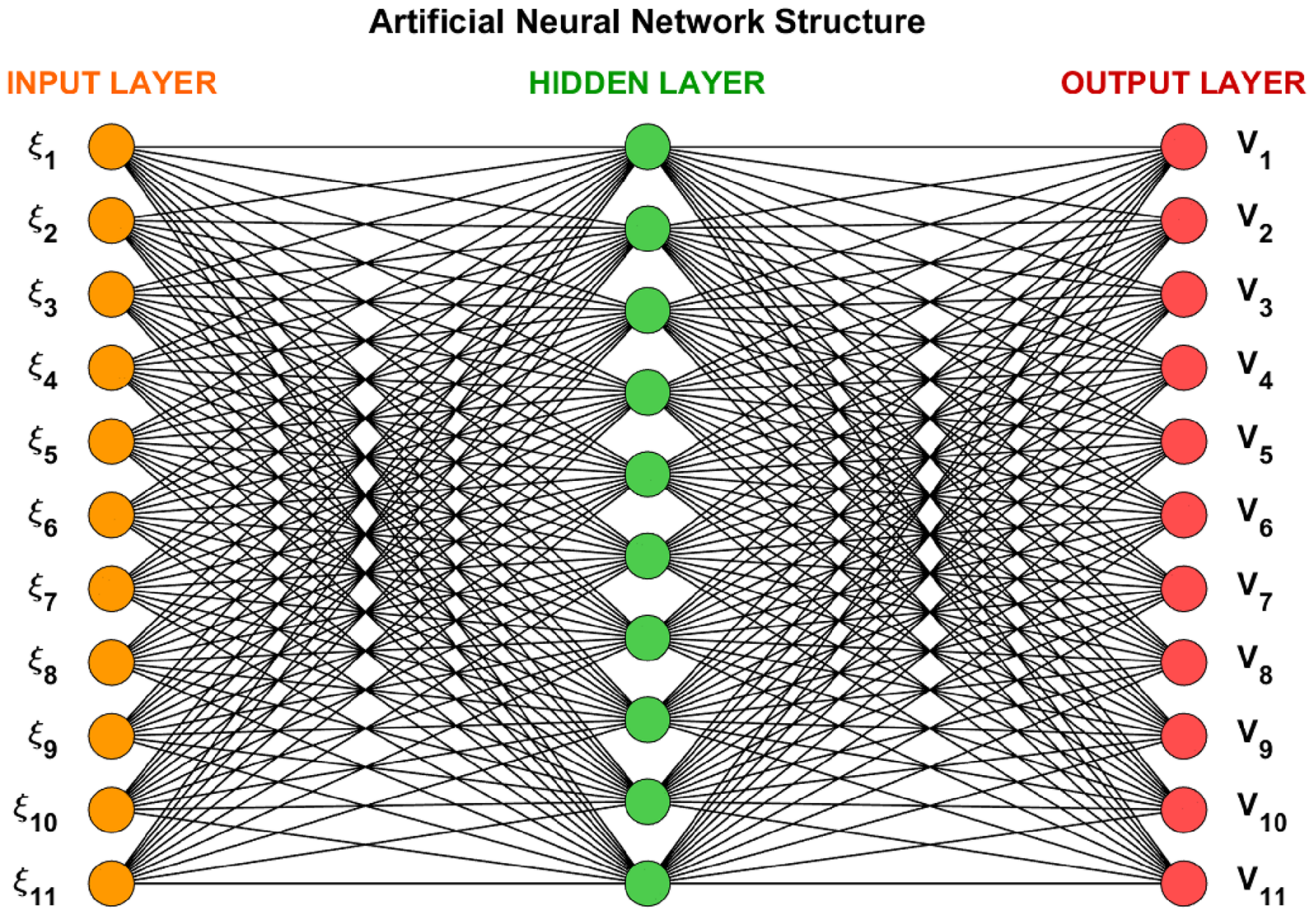
Artificial neural network structure.

## Results and discussion

In this study, employed the Legendre polynomial as an activation function in artificial neural networks (ANNs) and utilized a shallow neural network architecture to analyze the performance of the proposed model. The network are composed of 11 neurons in the input layer, 10 neurons in the hidden layer, and 11 neurons in the output layer, forming a balanced and computationally efficient configuration. The all results are adaptable biases and weights utilize in individually computation is 30, make sure to compromise the model solution accuracy and complexity illustrate in figure 2. Traditional activation functions such as ReLU, sigmoid and tanh reveal many constraint, such as restricted differentiability, gradient vanishing, and complexity in computing non-integer derivatives. To resolve these difficulties, the Legendre polynomial is acquaint this activation function because of its orthogonal models, mathematical tractability, and smooth differentiability, which optimized the fractional order model in computational technique.

The usage of Legendre the polynomial empower an uncomplicated, gives the stability and more accurate accomplishment of fractional-order derivatives, gives a significant advantage in modeling intricate physical problems. To further increases the network’s performance, engaged advanced optimization algorithms, including Particle Swarm Optimization (PSO), Fractional-Order Particle Swarm Optimization (FPSO), and their hybrid version (PSO–FPSO). The hybrid PSO–FPSO algorithm exhibited largest convergence and solution precise to compare the hybrid, PSO and FPSO techniques. This hybrid strategy impressively balances global search capabilities, minimizing the error between expected value and referenced material. The Legendre-based ANN optimized through PSO–FPSO represented maximum convergence, larger precision, and adaptability generalization attribute. The validity of this work is explored the proposed combined approach satisfies the constraint and shows extremely consistent with reference solutions, confirming its reliability and efficiency for complex computational analysis.

**Fig. 3:**
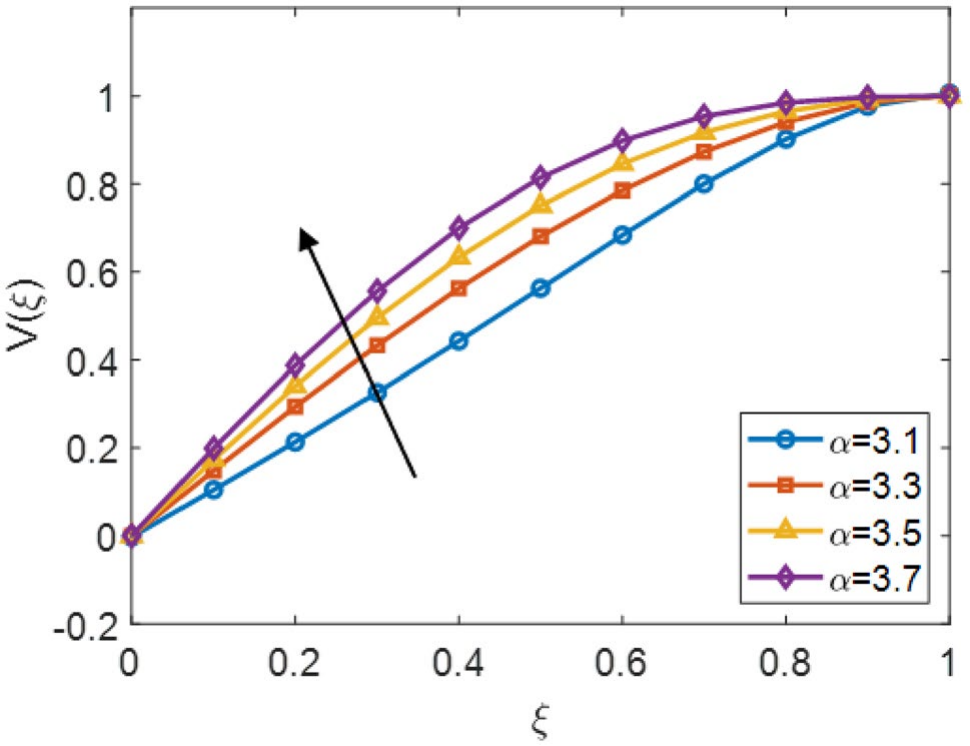
$V(\xi )$ for various ${\alpha }$ and $\mathrm {M_{p}}$ = 0.5, $\mathrm {M_{g}}$ = 0.5, $\mathrm {S_{q}}$ = - 0.3.

**Fig. 4:**
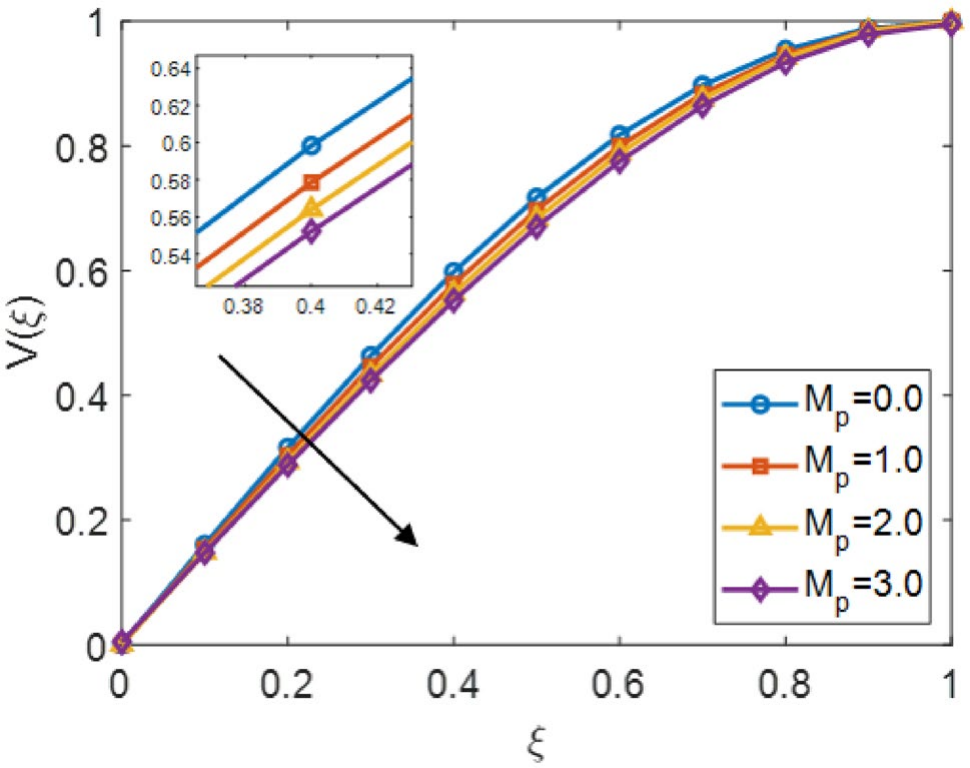
$V(\xi )$ for various $\mathrm {M_{p}}$ and $\alpha$ = 3.5, $\mathrm {M_{g}}$ = 0.5, $\mathrm {S_{q}}$ = 0.3.

The Fig. 3 shows the effects of the velocity distribution $V(\xi )$ with the similarity value $\xi$ for different values of the no-integer parameter $\alpha$ of Sutterby fluid. It is scrutiny that enhance the fractional order $\alpha$ for 3.1, 3.3, 3.5, and 3.7 paramount to intensify in the velocity profile through the flow domain. increasing values of $\alpha$ are related with higher velocity magnitudes and stronger acceleration, whereas minimum values of $\alpha$ express to decay velocity. Overall, the results highlight the vital role of the non-integer order parameter in controlling the velocity field and demonstrate the effectiveness of combining fractional calculus for modeling nonlocal and memory-dependent impacts in Sutterby fluid. This behavior is contained the memory effect induced by fractional order derivative, which allows to record the previous information of the fluid flow. Consequently, diffusion of fluid momentum is enhanced, which shows that the rises the fluid acceleration and gives the more optimal solution by compared with the prior model. The Fig. 4 illustrates the impacts of the velocity distribution $V(\xi )$ correspond the similarity variable $\xi$ for various values of the porosity parameter $M_p$ in the complex Sutterby fluid model. With the increasing of the $M_p$ parameter for 0.0, 1.0, 2.0 and 3.0, the velocity profile declines across the domain. This increasing permeability values shows that accelerates flow by the porous structure, which resists the fluid motion and decay behaviour of the velocity profile. The inset gives that the value of $\xi \approx 0.4$, showing the declining trend as $M_p$ rises. As a result, the non-integer model moderates the velocity restraint caused by the permeable values and the flow response indicate aggregated Previous states or conditions. larger fractional order model promote a smoother velocity distribution profile by refining the momentum transport through the fluid. This demonstrates that complex effects of the effectively balance porous resistance and provide a more realistic data of Sutterby fluid motion in permeable values.

**Fig. 5:**
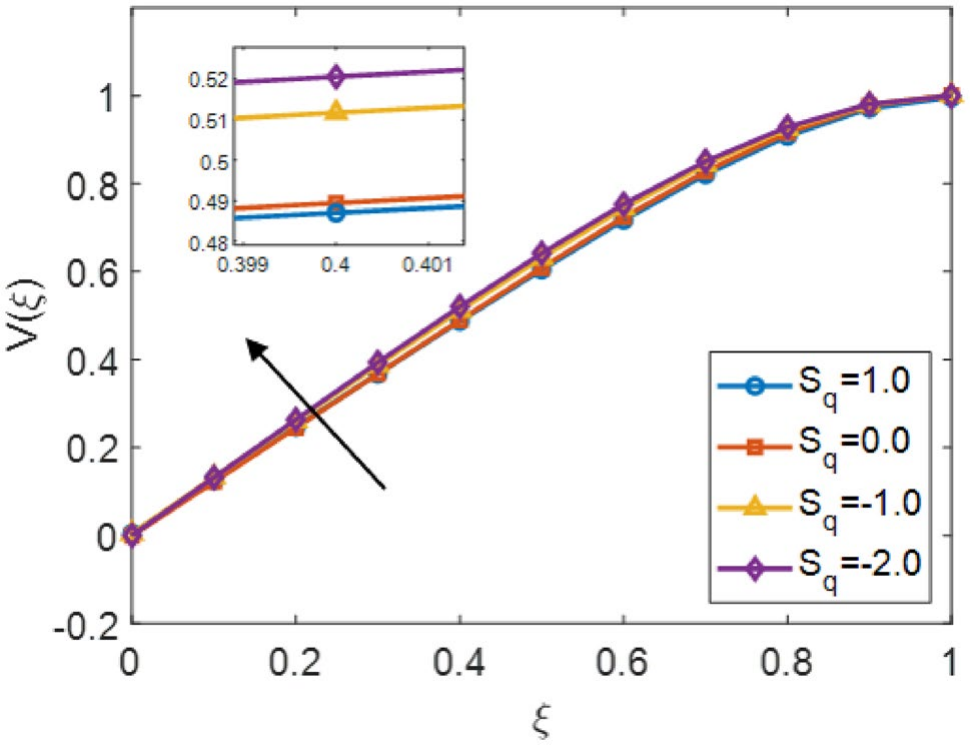
$V(\xi )$ for various $\mathrm {S_{q}}$ and $\alpha$ = 3.5, $\mathrm {M_{g}}$ = 0.5, $\mathrm {M_{p}}$ = 0.1.

**Fig. 6:**
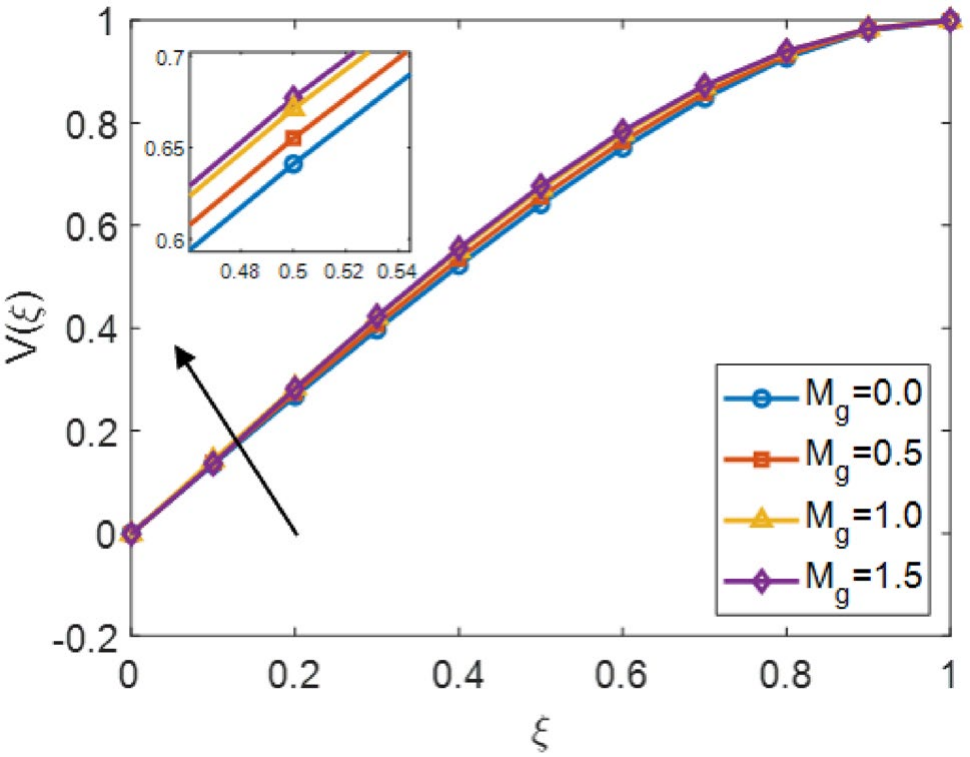
$V(\xi )$ for various $\mathrm {M_{g}}$ and $\alpha$ = 3.5, $\mathrm {M_{p}}$ = 0.1, $\mathrm {S_{q}}$ = 1.

The Fig. 5 shows the velocity distribution $V(\xi )$ correspond to the similarity variable $\xi$ for different values of the squeezing parameter $S_q$ in the fractional order Sutterby fluid. This figure illustrates that negative $S_q$ values $\{-1.0, -2.0\}$ lead to rises velocity, while positive $S_q$ values $\{0, 1.0\}$ result in a reduction in velocity profile. This represents that compressive squeezing resistive behaviour of the fluid motion and slows it, whereas stretching encourage fluid acceleration. The embedded values of $\xi \approx 0.4$ further indicates the opposite influence of negative and positive squeezing parameters. Overall, the squeezing parameter represents the control factor in determining whether the velocity profile of the fractional Sutterby fluid model is enhanced (intensified) or suppressed (restrain). Figure 6 represents the impacts of the magnetic variable $M_g$ on the velocity profile $V(\xi )$ of a fractional order Sutterby fluid. The velocity distribution rises with monotonically behaviour with $\xi$, and increasing values of $M_g$ elevate the profile, indicating that the magnetic force enlarged the fluid motion. The interaction between the fluid conductivity and magnetic field enhances momentum distribution, leading to adjust the profile upward in velocity curves.

**Fig. 7:**
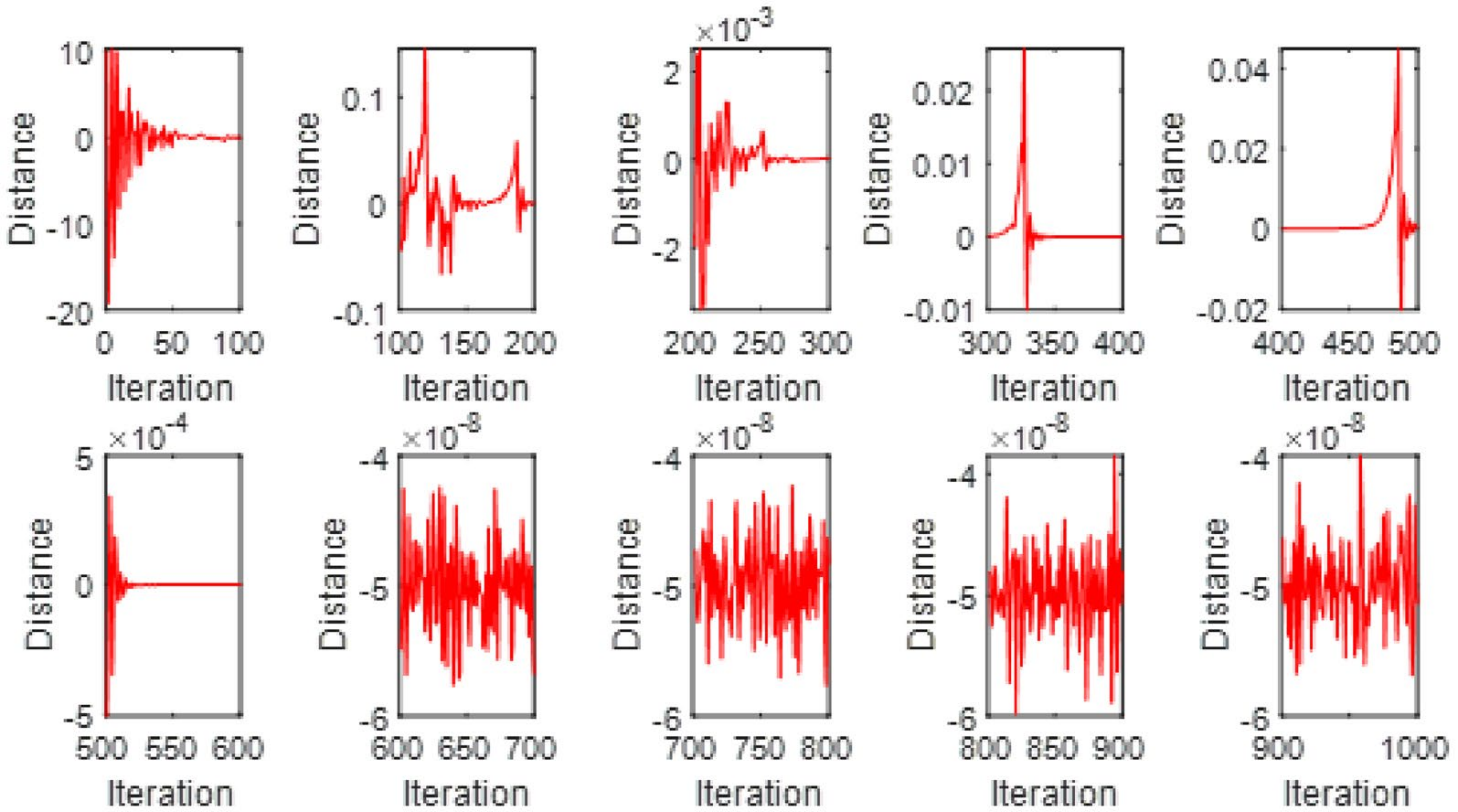
Swarm convergence for PSO-FPSO for case 1.

This Fig. 7 exhibits the convergence of the model behavior optimized through the Particle Swarm Optimization (PSO) technique. The subplots represents the changing of the error function with the number of iterations, emphasizing the training advance toward stabilization. The error illustrate noticeable oscillations, reflecting the difficulty of the optimizer in capturing the correct solution during early iterations. With further training, the error magnitude gradually declines, showing that the PSO achieves convergence with highly achievement of stability.

**Fig. 8:**
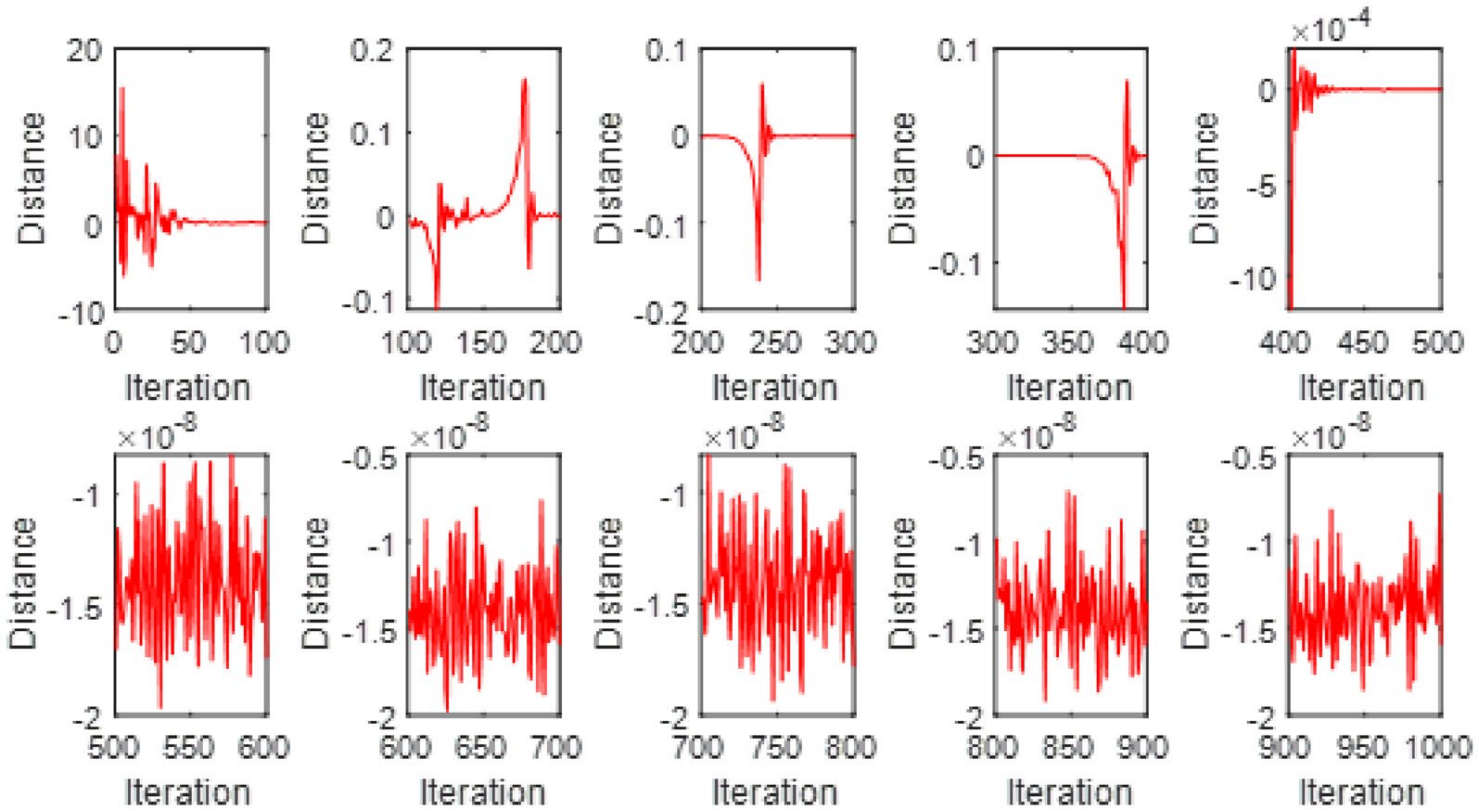
Swarm convergence for PSO-FPSO for case 2.

**Fig. 9:**
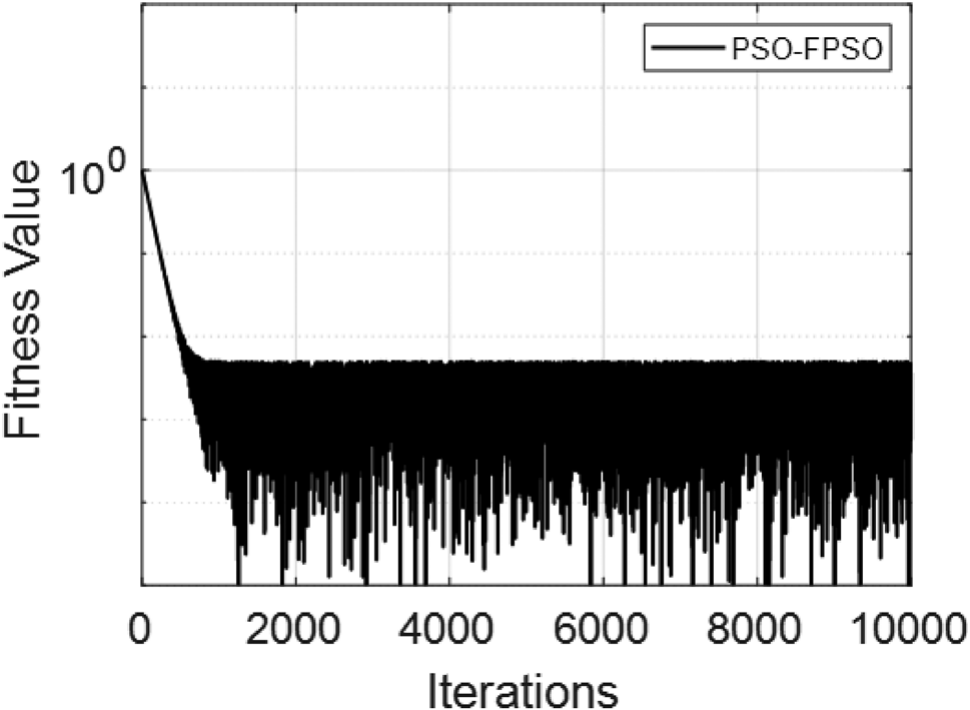
Fitness performance of the LANN through PSO-FPSO algorithm for case 1.

**Fig. 10:**
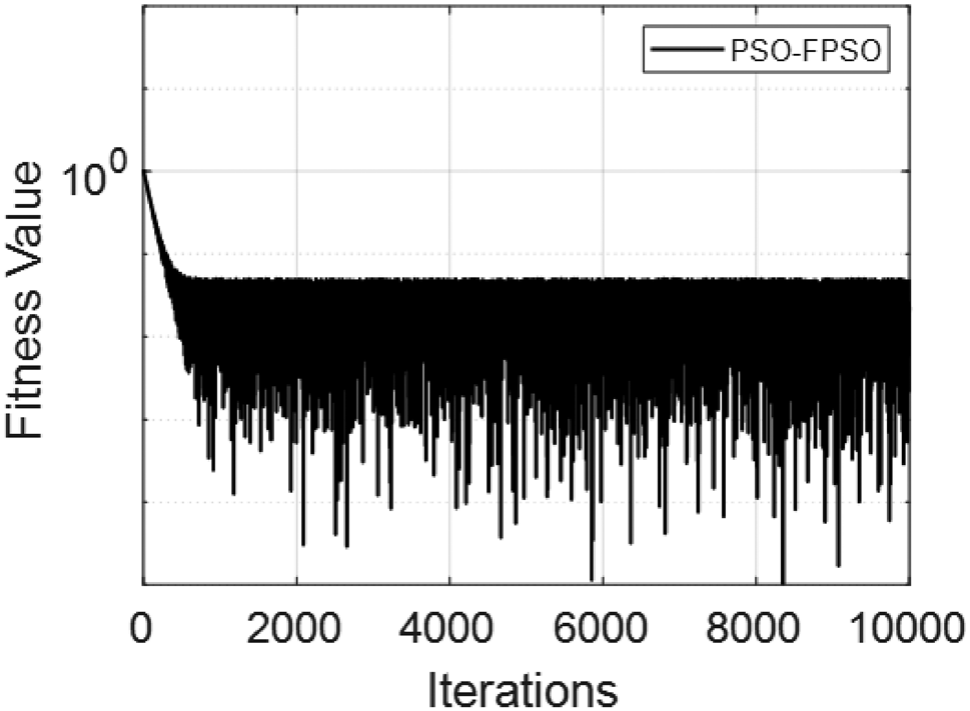
Fitness performance of the LANN through PSO-FPSO algorithm for case 2.

**Fig. 11:**
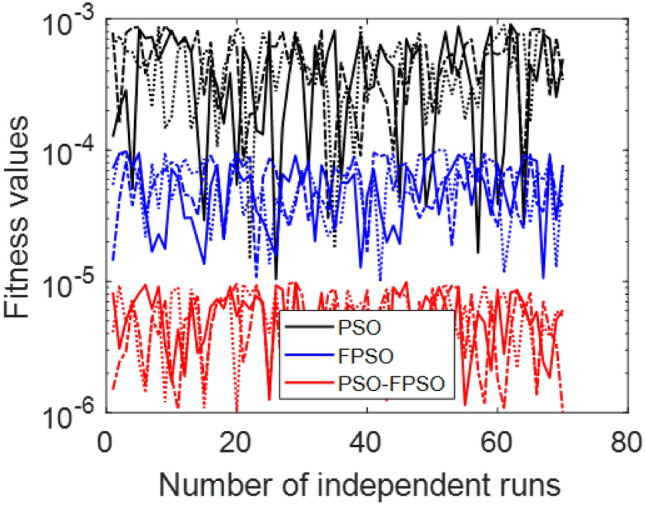
Convergence analysis of fitness function PSO, FPSO, and the hybrid PSO-FPSO for case 1.

**Fig. 12:**
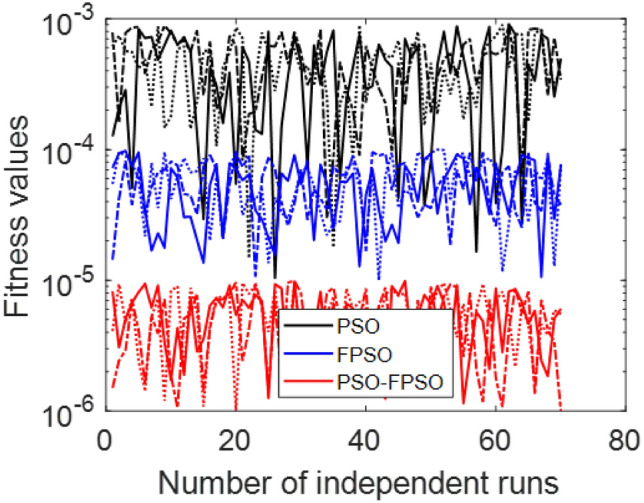
Convergence analysis of fitness function PSO, FPSO, and the hybrid PSO-FPSO for case 2.

The Fig. 8 presents the converging of the LANN, where the error is plotted with the number of iterations. The figure indicates that the error enlarge during training, at the start the iteration will be fluctuate and gradually approaching reliability and convergence. As iterations enhanced, the error residuals settle within a narrow range, representing that the model captures the underlying dynamics behaviours. This behavior accommodate that the applied optimizer make certain reliable training and effective convergence of the complex and non-integer Sutterby fluid. Figure 9 illustrates the convergent graph of the proposed combined technique in terms of fitness value versus iteration countable. The fitness values at the start extreme magnitude, reflecting the initial error in the approximation, indicating the strong global search capability of the algorithm. As the iterations progress, the fitness values oscillate and stabilize within a small band, showing the behaviour of the PSO-FPSO can be optimal or near-optimal solution. This convergence trend demonstrates the efficiency of the given method in achieving a minimized error while maintaining computational stability. Figure 10 represents the optimal value of the objective function of the hybrid algorithm for the nonlinear fractional order Sutterby model under the same computational technique. Similar to Fig. 9, a sharp reduction in the evaluation of the fitness function is noted at the beginning, which highlights the algorithm’s fast convergence. Beyond the initial phase, small fluctuations around the steady state are observed, reflecting the adaptive balance between exploration (searching new regions of the solution space) and exploitation (refining existing solutions). The near-zero error values obtained at higher iterations validate the accuracy, robustness, and credibility of the proposed approach for solving fractional nonlinear systems. Figures 11 and 12 indicates the convergence behavior of the evaluation of the fitness function for PSO, FPSO, and the hybrid of both for both Case 1 and Case 2. Conventional PSO achieves fitness values around $10^{-3}$, represents the lower convergence, while FPSO enhances this to approximately $10^{-4}$ due to non-integer order memory effects. The hybrid technique consistently reaches the lowest values $10^{-6}$, demonstrating faster convergence, higher accuracy, and more reliable performance across multiple independent runs. This improved convergence ensures precise solutions of the complex order differential equations, enhances the robustness, and reduces computational errors of the numerical modelling.

**Table 2:** Comparison of $V(\xi )$ for different values of ${\alpha }$ and $\mathrm {M_p}$.

$\mathrm {S_q}=-0.3, \mathrm {M_g}=0.5, \mathrm {M_p=0.5}$					$\mathrm {S_q}=-0.3, \mathrm {M_g}=0.5, \mathrm {M_p} =3.5$				
$\xi$	${\alpha =3.1}$	${\alpha }=3.3$	${\alpha }=3.5$	${\alpha }=3.7$	$\xi$	$\mathrm {M_p}=0$	$\mathrm {M_p}=1$	$\mathrm {M_p}=2$	$\mathrm {M_p}=3$
0.0	-0.0027329	-0.0005754	-4.42E−04	3.81E−05	0.0	0.0010116	0.0004181	5.90E−04	4.70E−03
0.1	0.1045696	0.1484285	0.1734939	0.1991695	0.1	0.1609219	0.1539437	0.1485052	0.1477112
0.2	0.2136450	0.2939755	0.3407807	0.3876075	0.2	0.3164748	0.3037644	0.2936146	0.2882812
0.3	0.3261329	0.4330326	0.4956863	0.5566778	0.3	0.4635529	0.4463890	0.4330766	0.4239661
0.4	0.4428632	0.5629317	0.6335835	0.7001510	0.4	0.5983534	0.5785768	0.5639981	0.5522397
0.5	0.5631568	0.6812218	0.7510400	0.8144053	0.5	0.7174640	0.6973410	0.6834253	0.6704213
0.6	0.6841546	0.7854839	0.8458775	0.8984770	0.6	0.8179588	0.7999338	0.7883354	0.7756017
0.7	0.8001767	0.8731098	0.9172012	0.9539962	0.7	0.8975152	0.8838138	0.8756278	0.8645624
0.8	0.9021110	0.9410437	0.9653999	0.9850106	0.8	0.9545506	0.9465952	0.9421156	0.9336903
0.9	0.9768307	0.9854879	0.9921153	0.9976953	0.9	0.9883807	0.9859786	0.9845173	0.9788859
1.0	1.0066423	1.0015710	1.0001822	0.9999490	1.0	0.9993970	0.9996640	0.9994487	0.9954667

The velocity profiles $V(\xi )$ shows in Tables 2 and 3 as the evaluations of the fitness functions for optimizing the Legendre-based Artificial Neural Network (LANN) framework. These values indicates the different combinations of non-integer order, micropolar and magnetic parameters that distinguish the rheological effects of the Sutterby fluid. The results illustrates that rises values of the fractional parameter ${\alpha }$ enhances the velocity profile, showing the stronger non-Newtonian effects and smoother convergence of the proposed neural network scheme. Conversely, higher values of the porosity parameter $\mathrm {M_p}$ and magnetic parameter $\mathrm {M_g}$ suppress the velocity field, revealing their resistive influence on flow motion and their role in stabilizing the learning process. Furthermore, the variation in the squeezing parameter $\mathrm {S_q}$ demonstrates that positive squeezing accelerates the fluid, contributing to faster minimization of the error function.

The LANN model accurately captures these nonlinear showing strong consistency, flow behaviors between the estimated and reference profiles. As the error term converges toward zero, the proposed approach achieves a precise approximation of the exact solution, verifying its computational reliability and efficiency. Overall, the integration of the complex fractional-order Sutterby model with the hybrid optimized technique models ensures rapid convergence, improved prediction accuracy, and minimized computational error. These findings shows that the constructed fitness functions are highly effective in determining the optimal biases and weights, thereby providing a efficient and robust tool for analyzing fractional non-Newtonian fluid flow dynamics.

**Table 3:** Comparison of $V(\xi )$ for different values of $\mathrm {S_q}$ and $\mathrm {M_g}$.

${\alpha }=3.5, \mathrm {M_g}=0.5, \mathrm {M_p=0.1}$					${\alpha }=3.5, \mathrm {S_q}=-0.1, \mathrm {M_p=0.1}$				
$\xi$	$\mathrm {S_q}=1$	$\mathrm {S_q}=0$	$\mathrm {S_q}=-1$	$\mathrm {S_q}=-2$	$\xi$	$\mathrm {M_g}=0$	$\mathrm {M_g}=0.5$	$\mathrm {M_g}=1$	$\mathrm {M_g}=1.5$
0.0	4.60E−03	−9.83E−04	4.38E−03	4.84E−05	0.0	−1.46E−04	−1.43E−03	−1.39E−03	−1.38E−03
0.1	0.1257902	0.1220154	0.1324554	0.1319273	0.1	0.1342510	0.1373675	0.1424684	0.1354487
0.2	0.2467842	0.2448443	0.2602523	0.2634113	0.2	0.2669542	0.2742473	0.2839117	0.2819140
0.3	0.3673829	0.3675070	0.3871136	0.3935407	0.3	0.3966655	0.4075073	0.4207970	0.4229167
0.4	0.4870384	0.4894735	0.5117069	0.5205601	0.4	0.5220101	0.5352707	0.5508008	0.5556574
0.5	0.6044442	0.6092039	0.6317863	0.6417921	0.5	0.6410766	0.6551585	0.6711737	0.6771032
0.6	0.7170742	0.7237265	0.7440007	0.7535743	0.6	0.7510507	0.7640691	0.7786287	0.7839826
0.7	0.8206689	0.8282696	0.8437471	0.8512590	0.7	0.8479417	0.8580625	0.8693607	0.8728468
0.8	0.9086679	0.9159487	0.9250693	0.9292756	0.8	0.9264042	0.9323497	0.9391995	0.9401976
0.9	0.9715888	0.9775080	0.9806013	0.9812562	0.9	0.9796523	0.9813871	0.9838949	0.9826814
1.0	0.9963531	1.0011169	1.0015559	1.0002232	1.0	0.9994676	0.9990770	0.9995343	0.9997350

## Conclusions

A fractional-order neural network scheme, constructed with Legendre polynomial approximations and optimized through PSO, FPSO, and a hybrid PSO–FPSO, has been employed to study the nonlinear unsteady Sutterby fluid flow. The method effectively handles the nonlinear and fractional-order nature of the governing equations, overcoming limitations of classical numerical techniques such as slow convergence and instability.

- The parametric investigation revealed that the fractional-order parameter enhances fluid acceleration through memory effects, permeability suppresses velocity due to porous resistance, the squeezing factor either promotes or resists motion depending on its sign, and magnetic influence consistently drives the flow upward.
- Convergence studies demonstrated that while standard PSO converges slowly with fluctuations, FPSO and especially the hybrid PSO–FPSO achieve faster, smoother, and more reliable optimization, with the hybrid method giving the most accurate outcomes.
- Overall, the proposed non integer-order ANN framework gives to be both robust and efficient, Sutterby fluid real life applications in polymer processing, lubricants, advanced industrial applications and, biomedical systems.

The limitation of my work is that only solved momentum equation, have not considered the heat transfer equation, concentration equations or any chemical reaction. The non-integer order model, which handles the memory effects, rises computational complexity and need to be judicious selection of ANN optimization and architecture parameters. Future research could expand the framework to incorporate thermal energy and nano-particles concentration effects, considered three-dimensional or curved surfaces, and investigate alternative fractional-order formulations or neural network architectures. These extensions would increased the applicability of the techniques and gives deeper understanding for practical engineering systems.

### Future work

We intend to utilize Morlet Wavelet Neural Networks (MWNNs) hybrid with other gradient-based algorithms namely, Genetic Algorithm (GA) and Water Cycle Algorithm (WCA) to extend the solution precision, validation and convergence performance.

## Supplementary Information

Supplementary Information.

## Data Availability

The datasets used or analysed during the current study available from the corresponding author on reasonable request.

## List of symbols

$u_{1}$: Velocity in *x*-direction ($\mathrm m\,\mathrm s^{-1}$)
$$\mu _{0}$$: Dynamic viscosity ($$\mathrm {kg \, m}^{-1}\,\mathrm s^{-1}$$)
$$\eta$$: Time variable (s)
*x*: Coordinate along the plate
$$\sigma$$: Electrical conductivity ($$\mathrm S\,\mathrm m^{-1}$$)
$$\rho$$: Fluid density ($${\rm kg}\,\mathrm m^{-3}$$)
$A_{1}$: First Rivlin–Ericksen tensor ($\mathrm s^{-1}$)
*B*: Characteristic time
$$M_{p}$$: Permeable factor
$v_{1}$: Velocity in *y*-direction ($\mathrm m\,\mathrm s^{-1}$)
$$\nu$$: Kinematic viscosity ($$\mathrm m^{2}\,\mathrm s^{-1}$$)
$$\xi$$: Stream function ($$\mathrm m^{2}\,\mathrm s^{-1}$$)
*y*: Coordinate normal to the plate
$$k^{*}$$: Permeability parameter ($$\mathrm m^{2}$$)
*k*: Thermal conductivity ($$\mathrm W\,\mathrm m^{-1}\,\mathrm K^{-1}$$)
$$S_{q}$$: Squeezing number
$${\bf V}$$: Velocity vector ($$\mathrm m\,\mathrm s^{-1}$$)
$$M_{g}$$: Magnetic parameter
