# Supplementary material for: Legendre neural network-based computational study through hybrid particle swarm optimization for fractional unsteady flow of Sutterby fluid
Source: Sci Rep. 2026 Apr 1;16:15285. doi: 10.1038/s41598-026-45305-7 (PMC13181085; doi:10.1038/s41598-026-45305-7)
Supplement: Supplementary file 1 — Supplementary Information. [file 41598_2026_45305_MOESM1_ESM.pdf]

Appendix: Legendre Polynomial-Based ANN (LANN) Solution Procedure

The non-integer order momentum equation model for Sutterby fluid through a surface, converted to an ordinary differential equation (ODE) by using similarity transformations. The solution space is emphasize estimation by a Legendre polynomial-based artificial neural network (LANN), where the independent variable denotes the ξ distance from the surface for the input and the velocity profile represents the output through graphically. The 6th-order Legendre polynomials ($n = 6$) structure utilized for the Perpendicular basis. The expression of the 6th-order polynomial is represents as:

$$P_6(\xi) = \frac{1}{16}\left(231\xi^6 - 315\xi^4 + 105\xi^2 - 5\right), \tag{1}$$

The apply of this orthogonal polynomial basis refines stability, decay in estimation error, and rises the numerical convergence, solving highly nonlinear fractional order differential equations. The network is trained in an unsupervised learning by minimizing the residual of the fractional ODE across selected collocation points. The fitness function is defined as the squared residual of the governing equation.

$$\begin{aligned}
e_1 = \Bigg[&\sum_{j=1}^{M}\left(1+\frac{mB^2}{2}\right)\sum_{i=1}^{N}\frac{\gamma_i}{a_1}\Big[720a_2\alpha_i^5(\alpha_i\xi+\beta_i)\Big] \\
&- M_p\sum_{i=1}^{N}\frac{\gamma_i}{a_1}\Big[30a_2\alpha_i^2(\alpha_i\xi+\beta_i)^4 - 12a_3\alpha_i^2(\alpha_i\xi+\beta_i)^2 + 2a_4\alpha_i^2\Big] \\
&- M_g\sum_{i=1}^{N}\frac{\gamma_i}{a_1}\Big[30a_2\alpha_i^2(\alpha_i\xi+\beta_i)^4 - 12a_3\alpha_i^2(\alpha_i\xi+\beta_i)^2 + 2a_4\alpha_i^2\Big] \\
&- S_q\Bigg\{\xi\sum_{i=1}^{N}\frac{\gamma_i}{a_1}\Big[a_2(\alpha_i\xi+\beta_i)^6 - a_3(\alpha_i\xi+\beta_i)^4 + a_4(\alpha_i\xi+\beta_i)^2 - a_5\Big] \\
&\quad + 3\sum_{i=1}^{N}\frac{\gamma_i}{a_1}\Big[30a_2\alpha_i^2(\alpha_i\xi+\beta_i)^4 - 12a_3\alpha_i^2(\alpha_i\xi+\beta_i)^2 + 2a_4\alpha_i^2\Big] \\
&\quad + \sum_{i=1}^{N}\frac{\gamma_i}{a_1}\Big[6a_2\alpha_i(\alpha_i\xi+\beta_i)^5 - 4a_3\alpha_i(\alpha_i\xi+\beta_i)^3 + 2a_4\alpha_i(\alpha_i\xi+\beta_i)\Big] \\
&\quad \times\sum_{i=1}^{N}\frac{\gamma_i}{a_1}\Big[30a_2\alpha_i^2(\alpha_i\xi+\beta_i)^4 - 12a_3\alpha_i^2(\alpha_i\xi+\beta_i)^2 + 2a_4\alpha_i^2\Big] \\
&\quad - \sum_{i=1}^{N}\frac{\gamma_i}{a_1}\Big[a_2(\alpha_i\xi+\beta_i)^6 - a_3(\alpha_i\xi+\beta_i)^4 + a_4(\alpha_i\xi+\beta_i)^2 - a_5\Big] \\
&\quad \times\sum_{i=1}^{N}\frac{\gamma_i}{a_1}\Big[120a_2\alpha_i^3(\alpha_i\xi+\beta_i)^3 - 24a_3\alpha_i^3(\alpha_i\xi+\beta_i)\Big]\Bigg\}\Bigg]^2.
\end{aligned}$$

$$
\begin{aligned}
e_2 = \frac{1}{4}\sum_{j=1}^{M}\Bigg[&\left(\sum_{i=1}^{N}\frac{\gamma_i}{a_1}\Big[a_2(\alpha_i(0)+\beta_i)^6 - a_3(\alpha_i(0)+\beta_i)^4 + a_4(\alpha_i(0)+\beta_i)^2 - a_5\Big]\right)^2 \\
&+\left(\sum_{i=1}^{N}\frac{\gamma_i}{a_1}\Big[6a_2\alpha_i(\alpha_i\cdot 1+\beta_i)^5 - 4a_3\alpha_i(\alpha_i\cdot 1+\beta_i)^3 + 2a_4\alpha_i(\alpha_i\cdot 1+\beta_i)\Big]\right)^2 \\
&+\left(\sum_{i=1}^{N}\frac{\gamma_i}{a_1}\Big[30a_2\alpha_i^2(\alpha_i(0)+\beta_i)^4 - 12a_3\alpha_i^2(\alpha_i(0)+\beta_i)^2 + 2a_4\alpha_i^2\Big]\right)^2 \\
&+\left(\sum_{i=1}^{N}\frac{\gamma_i}{a_1}\Big[6a_2\alpha_i(\alpha_i(1)+\beta_i)^5 - 4a_3\alpha_i(\alpha_i(1)+\beta_i)^3 + 2a_4\alpha_i(\alpha_i(1)+\beta_i)\Big] - 1\right)^2\Bigg].
\end{aligned}
$$

To optimize the ANN weights, a hybrid PSO–FPSO algorithm is applied, combining the global search capability of classical particle swarm optimization with the enhanced exploitation mechanism provided by fractional-order updating. Convergence is accomplish when the residuum achieve the specified tolerance range. This method gives the efficient and accurate computation model of the velocity distribution for the fractional order Sutterby fluid under differnt flow conditions and non-integer parameters.

$$
\begin{aligned}
&\hat{V}(\xi) = \sum_{i=1}^{10}\frac{1}{a_1}\gamma_i\left[a_2(\alpha_i\xi+\beta_i)^6 - a_3(\alpha_i\xi+\beta_i)^4 + a_4(\alpha_i\xi+\beta_i)^2 - a_5\right], \\
&where, a_1 = 16, \quad a_2 = 0.001, \quad a_3 = 0.01, \quad a_4 = 0.003, \quad a_5 = 5,
\end{aligned}
$$

Case 1:

$$
\begin{aligned}
\hat{V}(\xi) \;=\; & \frac{1}{16}(-6.1903)\left[0.001(2.181\xi-1.685)^6 - 0.01(2.181\xi-1.685)^4 + 0.003(2.181\xi-1.685)^2 - 5\right] \\
&+\frac{1}{16}(3.052)\left[0.001(-1.201\xi+0.562)^6 - 0.01(-1.201\xi+0.562)^4 + 0.003(-1.201\xi+0.562)^2 - 5\right] \\
&+\frac{1}{16}(5.108)\left[0.001(1.875\xi-0.003)^6 - 0.01(1.875\xi-0.003)^4 + 0.003(1.875\xi-0.003)^2 - 5\right] \\
&+\frac{1}{16}(-2.992)\left[0.001(0.572\xi-1.579)^6 - 0.01(0.572\xi-1.579)^4 + 0.003(0.572\xi-1.579)^2 - 5\right] \\
&+\frac{1}{16}(0.074)\left[0.001(-1.772\xi-1.811)^6 - 0.01(-1.772\xi-1.811)^4 + 0.003(-1.772\xi-1.811)^2 - 5\right] \\
&+\frac{1}{16}(-1.443)\left[0.001(1.937\xi-5.667)^6 - 0.01(1.937\xi-5.667)^4 + 0.003(1.937\xi-5.667)^2 - 5\right] \\
&+\frac{1}{16}(1.367)\left[0.001(-2.728\xi+4.886)^6 - 0.01(-2.728\xi+4.886)^4 + 0.003(-2.728\xi+4.886)^2 - 5\right] \\
&+\frac{1}{16}(-2.596)\left[0.001(-2.311\xi+1.652)^6 - 0.01(-2.311\xi+1.652)^4 + 0.003(-2.311\xi+1.652)^2 - 5\right] \\
&+\frac{1}{16}(-1.9702)\left[0.001(0.488\xi+3.834)^6 - 0.01(0.488\xi+3.834)^4 + 0.003(0.488\xi+3.834)^2 - 5\right] \\
&+\frac{1}{16}(0.841)\left[0.001(1.151\xi+0.083)^6 - 0.01(1.151\xi+0.083)^4 + 0.003(1.151\xi+0.083)^2 - 5\right]
\end{aligned}
$$

Case 2:

$$
\begin{aligned}
\hat{V}(\xi) \;=\; & \frac{1}{16}(-5.498)\left[0.001(2.092\xi-0.825)^6-0.01(2.092\xi-0.825)^4+0.003(2.092\xi-0.825)^2-5\right]\\
&+\frac{1}{16}(3.121)\left[0.001(-2.321\xi+0.704)^6-0.01(-2.321\xi+0.704)^4+0.003(-2.321\xi+0.704)^2-5\right]\\
&+\frac{1}{16}(5.414)\left[0.001(2.221\xi+0.031)^6-0.01(2.221\xi+0.031)^4+0.003(2.221\xi+0.031)^2-5\right]\\
&+\frac{1}{16}(-3.485)\left[0.001(-1.436\xi-1.611)^6-0.01(-1.436\xi-1.611)^4+0.003(-1.436\xi-1.611)^2-5\right]\\
&+\frac{1}{16}(-0.09)\left[0.001(-2.027\xi+0.412)^6-0.01(-2.027\xi+0.412)^4+0.003(-2.027\xi+0.412)^2-5\right]\\
&+\frac{1}{16}(-1.601)\left[0.001(1.827\xi-5.166)^6-0.01(1.827\xi-5.166)^4+0.003(1.827\xi-5.166)^2-5\right]\\
&+\frac{1}{16}(1.782)\left[0.001(-3.232\xi+3.898)^6-0.01(-3.232\xi+3.898)^4+0.003(-3.232\xi+3.898)^2-5\right]\\
&+\frac{1}{16}(-2.589)\left[0.001(-1.618\xi+0.826)^6-0.01(-1.618\xi+0.826)^4+0.003(-1.618\xi+0.826)^2-5\right]\\
&+\frac{1}{16}(-1.521)\left[0.001(2.029\xi-0.003)^6-0.01(2.029\xi-0.003)^4+0.003(2.029\xi-0.003)^2-5\right]\\
&+\frac{1}{16}(1.123)\left[0.001(0.532\xi-0.057)^6-0.01(0.532\xi-0.057)^4+0.003(0.532\xi-0.057)^2-5\right]
\end{aligned}
$$
